# Developmental vitamin D and autism spectrum disorders: findings from the Stockholm Youth Cohort

**DOI:** 10.1038/s41380-019-0578-y

**Published:** 2019-11-06

**Authors:** Brian K. Lee, Darryl W. Eyles, Cecilia Magnusson, Craig J. Newschaffer, John J. McGrath, David Kvaskoff, Pauline Ko, Christina Dalman, Håkan Karlsson, Renee M. Gardner

**Affiliations:** 1 Department of Epidemiology and Biostatistics, Drexel University School of Public Health, Philadelphia, PA USA; 2 A.J. Drexel Autism Institute, Philadelphia, PA USA; 3 Department of Public Health Sciences, Karolinska Institutet, Stockholm, Sweden; 4 Queensland Brain Institute, University of Queensland, St. Lucia, QLD Australia; 5 Queensland Centre for Mental Health Research, The Park Centre for Mental Health, Wacol, QLD Australia; 6 National Centre for Register-based Research, Aarhus BSS, Aarhus University, Aarhus, Denmark; 7 Department of Neuroscience, Karolinska Institutet, Stockholm, Sweden

**Keywords:** Autism spectrum disorders, Predictive markers

## Abstract

Animal studies indicate that early life vitamin D is crucial for proper neurodevelopment. Few studies have examined whether maternal and neonatal vitamin D concentrations influence risk of autism spectrum disorders (ASD). Participants were sampled from the Stockholm Youth Cohort, a register-based cohort in Sweden. Concentrations of total 25-hydroxyvitamin D (25OHD) were assessed from maternal and neonatal biosamples using a highly sensitive liquid chromatography tandem mass spectrometry method. The maternal sample consisted of 449 ASD cases and 574 controls, the neonatal sample: 1399 ASD cases and 1607 controls; and the paired maternal-neonatal sample: 340 ASD cases and 426 controls. Maternal 25OHD was not associated with child ASD in the overall sample. However, in Nordic-born mothers, maternal 25OHD insufficiency (25 − <50 nmol/L) at ~11 weeks gestation was associated with 1.58 times higher odds of ASD (95% CI: 1.00, 2.49) as compared with 25OHD sufficiency (≥50 nmol/L). Neonatal 25OHD < 25 nmol/L was associated with 1.33 times higher odds of ASD (95% CI: 1.02, 1.75) as compared with 25OHD ≥ 50 nmol/L. Sibling-matched control analyses indicated these associations were not likely due to familial confounding. Children with both maternal 25OHD and neonatal 25OHD below the median had 1.75 (95% CI: 1.08, 2.86) times the odds of ASD compared with children with maternal and neonatal 25OHD both below the median. Our results are consistent with an increasing body of evidence suggesting that vitamin D concentrations in early life may be associated with increased risk of neurodevelopmental disorders including ASD.

## Introduction

Early life nutrition is critical for proper neurodevelopment [1] and the paradigm may extend to autism spectrum disorders (ASD) as well [2]. Epidemiological studies have suggested potential links between multiple nutritional factors and ASD risk, including antenatal folic acid [3] or multivitamin supplementation [4], fat intake [5], and iron intake [6], although findings are not consistent across studies [7, 8]. One nutritional factor of potential relevance to ASD risk is vitamin D [9]. Although commonly referred to as the “sunshine vitamin”, vitamin D is a neuroactive hormone that is required for normal brain homeostasis and neurodevelopment [10]. Most (~90%) vitamin D is synthesized in response to skin exposure to UV-B light with the rest derived from dietary sources [11]. Vitamin D deficiency and insufficiency are common globally, especially in pregnant women [12, 13], and are more prevalent in winter, in high latitudes, urban settings and in dark-skinned individuals [14, 15].

Recent epidemiological studies have provided evidence that lower concentrations of gestational 25-hydroxyvitamin D (25OHD) may be correlated with increased risk of ASD phenotypes. In an Australian birth cohort, maternal mid-gestation 25OHD insufficiency was associated with increased offspring risk of high scores on the Attention Switching subscale of the Autism Spectrum Quotient [16] and increased risk of language impairment [17]. In the Generation R study in the Netherlands, lower mid-gestation and cord blood 25OHD concentrations were associated with more abnormal scores on the Social Responsiveness Scale, a measure of an ASD-related phenotype [18]. There is also evidence suggesting that early life 25OHD may be associated with ASD case status, although findings have been mixed. Two studies—one of Swedish siblings with ASD and their unaffected siblings (*n* = 58 pairs) and a Chinese ASD case-control study (*n* = 68 cases)—found that lower levels of neonatal [19] and 1st trimester 25OHD [20] were associated with increased odds of ASD. In the Generation R study (also based on 68 cases), mid-gestation vitamin D deficiency was associated with 2.4 times higher risk of ASD [21]. However, two recent studies in California found that neonatal 25OHD was not associated with ASD overall, although there was some evidence of associations in subgroups (see Discussion) [22, 23].

In the present study, we examine the associations of maternal and neonatal 25OHD and later risk of ASD in a Swedish population-based sample. Because of prior reported associations of early life 25OHD with intellectual function, we also explored whether associations were different for ASD subtyped by presence or absence of co-occurring intellectual disability (ID).

## Methods

### Study sample

Ethical approval was provided by the regional ethical review board for Karolinska Institutet (DNR 2010/1185-31/5). As this was a register-based study, no individual level consent was required. All data were anonymized before being received by the research team. The Stockholm Youth Cohort (SYC) is a register-based cohort of all children aged 0–17 years living in Stockholm County. The source population for the present study was the 98,597 SYC participants born in Sweden 1996–2000 and resident for at least 4 years in Stockholm County through 2011. The length of residency exclusion criteria is to allow a reasonable opportunity for local healthcare services to detect ASD.

There were four analytic samples based on the available biospecimens (Figs. S1 and S2): the maternal sample examining prenatal 25OHD in maternal sera; the neonatal sample examining neonatal 25OHD in dried blood spots (DBS) taken soon after birth; the neonatal sibling sample examining neonatal 25OHD in cases matched to unaffected siblings; and the paired maternal-neonatal sample examining both maternal and neonatal 25OHD (restricted to persons with both maternal and neonatal 25OHD measures).

### ASD diagnoses

Developmental screenings are carried out free of charge in Stockholm County at regular intervals from 1 to 60 months by a pediatrician or nurse. With a positive screen, children are referred to specialist services for formal diagnostic assessment. We implemented a register-based case-finding procedure covering all pathways to care in Stockholm County. Briefly, ASD status captured by ICD-9 (299), ICD-10 (F84), and DSM-IV (299) codes using the National Patient Register, Habilitation Register, the Clinical Database for Child and Adolescent Psychiatry, and the VAL database covering all inpatient and outpatient health services use in Stockholm County. Ascertainment of ID was based on ICD-9 (317–319), ICD-10 (F70-F79), and DSM-IV (317–319) codes and supplemented with information in the Habilitation Register, which classifies services recipients as having ID or not. In a validation study, we found case-notes consistent with an ASD diagnosis for 96.0% of the register-identified cases [24].

### 25OHD analysis

Since 1975 in Sweden, DBS have been collected from all newborns at ~3–5 days of age and stored at a central biobank for metabolic disease screening. DBS were obtained for each participant, and 3.2 mm punches were extracted and derivatized in a process optimized for detection of vitamin D [25]. For the maternal sample, sera were obtained at median 10.9 weeks gestation (IQR: 9.3, 13.0). In total, 78.0% of sera samples were from the 1st trimester, 19.0% from the 2nd, and 3.0% from the 3rd trimester. Because of the range of gestational ages at which sera was collected, and because 25OHD varies across the calendar year (i.e., seasonal variation) and during pregnancy, 25OHD values are not directly comparable between participants if, for example, one participant had a 3rd trimester 25OHD measurement while another participant had a 1st trimester measurement. Consistent with recent practice [26], we used cosinor modeling to standardize 25OHD values at gestational age of 10.9 weeks (Supplement Fig. S3).

25OHD was measured using a tandem mass spectrometry assay developed by DWE [25], including recent methods to improve sensitivity to 100 pmol/L in DBS [27]. This assay separately measures the endogenous 25OHD3 and any exogenous 25OHD2 resulting from maternal supplementation. This assay has been used in a number of studies exploring the association between 25OHD with autism and schizophrenia [18, 21–23, 28, 29]. The analytic variable of interest was the concentration of total 25OHD (25OHD3 + 25OHD2). These values largely represent the measurement of 25OHD3, as few serum (0.2%) or DBS samples (0.4%) had detectable levels of 25OHD2. Because the neonatal samples were obtained from venous blood, the following correction formula was used to estimate equivalent serum levels: (DBS 25OHD3 + DBS 25OHD2) × (1/(1–0.55)), where the 0.55 is a reference hematocrit value for venous blood [25, 30–32].

To assess construct validity of the 25OHD measures, we assessed (1) whether there was seasonal variation in 25OHD depending on month of collection and (2) whether 25OHD measures correlated with sunlight intensity around the time of birth. Sunlight measurements for Stockholm during the study period (specifically, average insolation incident on a horizontal surface) were obtained from the NASA Prediction of Worldwide Energy Resource [33]. To standardize comparisons, 25OHD values and solar radiation values were z-transformed and then plotted against each other.

### Covariates

In addition to the aforementioned health registers, covariate data were extracted from the Medical Birth Register and the Integrated Database for Labor Market Research. Covariates were identified a priori from the literature as being associated with either vitamin D or ASD status. Year and season of birth were always included to account for calendar time and seasonal trends [34]. Other covariates considered included diagnoses of maternal autism, ID, and other psychiatric disorders [35] and maternal health variables assessed at the first antenatal visit, including body mass index [36], smoking [37], and nutritional supplement use [4]. Gestational age at birth was included to account for the increased risk of ASD observed for non-term births [38]. Although race/ethnicity data were not available, maternal region of origin (Nordic, other Europe, Africa, Asia, other) was included in models.

### Statistical analysis

The outcomes of interest were ASD, ASD without co-occurring ID, and ASD with ID. Logistic regression models were used to estimate odds ratios and 95% confidence intervals (OR, 95% CI). 25OHD was analyzed with the following categorizations: deficient (<25 nmol/L), insufficient (25 − <50 nmol/L), and sufficient (≥50 nmol/L), consistent with recommendations set by the Institute of Medicine [39]. To check for potential threshold or non-linear associations, penalized cubic regression smoothing splines in generalized additive models were implemented to assess 25OHD as a continuous variable. We examined possible effect modification of the associations of neonatal vitamin D and ASD by child sex and by maternal region of origin as a proxy for skin pigmentation. For the sibling-matched sample, conditional logistic regression was employed to account for the matched nature of the analysis. Because siblings share genetics and early life environment, this analysis can test whether results from the standard analyses may be due to confounding from these sources.

Biosamples were randomly plated to avoid the possibility of systematic confounding due to plate effects. To maintain the blinded status of lab personnel as well as participant integrity, unaffected siblings were plated separately from their matched cases. Therefore, the plate-specific 25OHD *z*-score (calculated as the number of standard deviations away from the plate-specific mean a particular observation is) was used for the matched sibling analysis. All analyses were conducted using R 3.4.1 [40]. R code for analysis will be made available upon request.

Although predictive modeling for standardization of 25OHD is common, inferences resulting from this modeling may be incorrect if the underlying predictive model used to standardize the values is incorrect. We conducted a sensitivity analysis restricted to the 78% of participants with 25OHD values measured in the 1st trimester, using the natural 25OHD values instead of the standardized 25OHD values.

## Results

### Study sample

The maternal sample consisted of 449 ASD cases and 574 controls; the neonatal sample, 1399 ASD cases and 1607 controls; the neonatal sibling sample, 357 ASD cases matched to 364 unaffected siblings; and the paired maternal-neonatal sample, 340 ASD cases and 426 controls. Because the samples were broadly similar, the neonatal sample is described here, and the other samples are described in Table S1. Of the neonatal controls, 25OHD-sufficient participants were more likely to have mothers who took multivitamin supplements, who had a normal BMI, and who were born in a Nordic country, compared with insufficient or deficient participants (Table 1). Persons excluded due to non-collection of samples and laboratory failures differed from persons included in the sample on some aspects. They were more likely to be born in spring; have missing maternal BMI; less maternal prenatal iron only supplement use; and less likely to have maternal origin in Nordic countries (Table S2).

**Table 1:** Neonatal analytic sample characteristics

	By case status		By 25OHD status (No ASD only)		
	No ASD: *n* = 1607	ASD: *n* = 1399	25OHD Sufficient ≥ 50 nmol/L, *n* = 186	25OHD: Insufficient 25 − <50 nmol/L, *n* = 682	25OHD: Deficient <25 nmol/L, *n* = 739
Outcome					
ASD no ID	–	947 (67.7)	–	–	–
ASD with ID	–	452 (32.3)	–	–	–
No ASD	1607 (100)		186 (100)	682 (100)	739 (100)
Season of birth					
Spring (Mar–May)	407 (25.3)	304 (21.6)	22 (11.8)	148 (21.7)	237 (32.1)
Summer (Jun–Aug)	436 (27.1)	405 (28.9)	103 (55.4)	193 (28.3)	140 (18.9)
Fall (Sep–Nov)	391 (24.3)	376 (26.9)	47 (25.3)	185 (27.1)	159 (21.5)
Winter (Dec–Feb)	373 (23.2)	314 (22.4)	14 (7.5)	156 (22.9)	203 (27.5)
Male	819 (51.0)	1064 (76.1)	85 (45.7)	365 (53.5)	369 (49.9)
Maternal age, mean (SD)	30.2 (5.1)	30.0 (5.3)	30.7 (4.6)	30.8 (4.9)	29.5 (5.2)
Maternal BMI					
Normal	828 (51.5)	623 (44.5)	107 (57.5)	348 (51.0)	373 (50.5)
Underweight	38 (2.4)	37 (2.6)	2 (1.1)	17 (2.5)	19 (2.6)
Overweight	262 (16.3)	260 (18.6)	20 (10.8)	108 (15.8)	134 (18.1)
Obese	88 (5.5)	104 (7.4)	1 (0.5)	37 (5.4)	50 (6.8)
Missing	391 (24.3)	375 (26.8)	56 (30.1)	172 (25.2)	163 (22.1)
Maternal smoking	172 (10.7)	158 (11.3)	16 (8.6)	69 (10.1)	87 (11.8)
Maternal supplementation					
Multivitamins	201 (12.5)	166 (11.9)	33 (17.7)	105 (15.4)	63 (8.5)
Iron only	498 (31.0)	456 (32.6)	44 (23.7)	203 (29.8)	251 (34.0)
Folic acid only	13 (0.8)	20 (1.4)	2 (1.1)	7 (1.0)	4 (0.5)
Iron and folic acid	142 (8.8)	104 (7.4)	8 (4.3)	52 (7.6)	82 (11.1)
None of the above	750 (46.7)	653 (46.7)	98 (52.7)	315 (46.2)	337 (45.6)
Maternal psychiatric history	550 (34.2)	694 (49.6)	53 (28.5)	229 (33.6)	268 (36.3)
Maternal birth country					
Nordic	1243 (77.3)	1079 (77.1)	177 (95.2)	619 (90.8)	447 (60.5)
Africa	90 (5.6)	97 (6.9)	0 (0)	4 (0.6)	86 (11.6)
Asia	166 (10.3)	112 (8.0)	2 (1.1)	17 (2.5)	147 (19.9)
Other Europe	66 (4.1)	57 (4.1)	4 (2.2)	26 (3.8)	36 (4.9)
Other	42 (2.6)	54 (3.9)	3 (1.6)	16 (2.3)	23 (3.1)

### Vitamin D measures

**Fig. 1:**
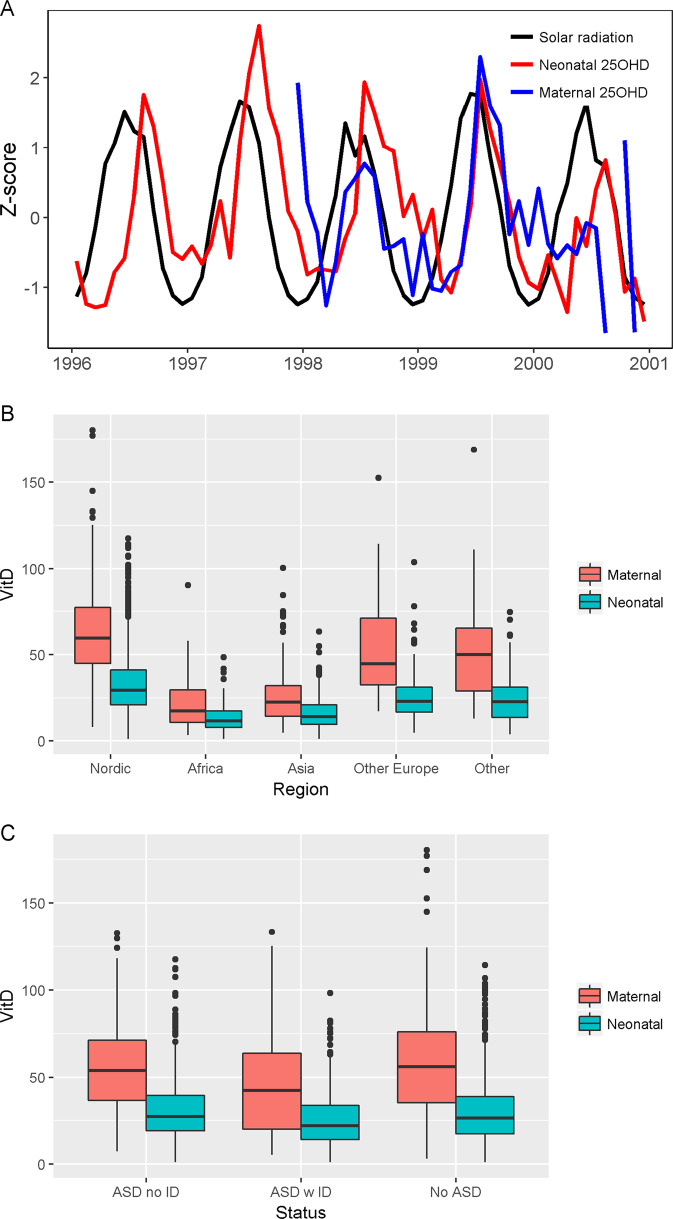
Neonatal and maternal (natural) 25OHD concentrations by time of sampling (**a**), maternal region of origin (**b**), and ASD case status (**c**)

25OHD concentrations by case status and maternal region of origin are summarized in Table 2. Median (IQR) values of standardized maternal and neonatal 25OHD were 57.1 nmol/L (45.8, 65.1) and 26.1 (17.4, 38.2), respectively. Of the mothers, 67.5% had sufficient 25OHD, 23.9% insufficient 25OHD, and 8.6% deficient 25OHD. Of the neonates, 10.0% had sufficient 25OHD, 41.2% insufficient 25OHD, and 48.7% deficient 25OHD. Maternal and neonatal were moderately correlated (Spearman correlations for maternal natural 25OHD and neonatal 25OHD: 0.50; and maternal standardized 25OHD and neonatal 25OHD: 0.45). Figure 1a shows a seasonal pattern such that higher 25OHD values are observed for summer births and lower values for winter births. 25OHD measures varied by maternal region of origin, such that Nordic-born mothers had the highest concentrations and Africa-born mothers the lowest (Fig. 1b).

**Table 2:** Median (IQR) of 25OHD concentrations in nmol/L

	Maternal 25OHD (natural)	Maternal 25OHD (standardized to 10.9 weeks gestation)	Neonatal 25OHD
Entire sample	54.2 (33.9, 73.1)	57.1 (45.8, 65.1)	26.1 (17.4, 38.2)
Nordic-born mothers	59.5 (45.0, 77.4)	60.0 (53.0, 68.2)	29.3 (20.8, 41.2)
Non-Nordic born mothers	26.7 (15.6, 44.2)	30.8 (22.7, 45.1)	15.1 (9.8, 23.0)
By outcome status (entire sample)			
ASD	50.2 (32.6, 68.4)	55.1 (45.7, 64.0)	25.7 (17.5, 37.6)
ASD with ID	43.8 (19.7, 63.8)	49.7 (28.7, 63.2)	22.7 (14.3, 34.1)
ASD without ID	54.2 (36.5, 69.7)	57.2 (49.0, 64.2)	26.9 (18.8, 39.2)
No ASD	56.1 (35.1, 76.3)	58.1 (46.3, 66.5)	26.6 (17.3, 38.6)
By outcome status (Nordic-born mothers)			
ASD	56.4 (42.5, 73.7)	58.9 (51.7, 66.9)	28.9 (20.6, 40.7)
ASD with ID	57.7 (43.8, 73.8)	59.8 (51.7, 68.5)	29.3 (20.7, 41.0)
ASD without ID	56.1 (42.4, 73.3)	58.8 (51.8, 65.7)	28.7 (20.6, 40.5)
No ASD	62.3 (47.6, 79.6)	60.8 (54.3, 68.7)	29.7 (21.0, 42.2)

Number of maternal samples = 1023; Number of neonatal samples = 3006

### Maternal vitamin D and ASD

For standardized maternal 25OHD, persons without ASD had median (IQR) concentrations of 56.1 nmol/L (35.1, 76.3), as compared to persons with ASD with ID [43.8 nmol/L (19.7, 63.8)] and ASD without ID [54.2 nmol/L (36.5, 69.7)] (Table 2, Fig. 1c).

In adjusted models, there were no associations of maternal 25OHD with child ASD in the total sample (Table 3). In the subsample of participants with Nordic-born mothers, those with insufficient maternal 25OHD had 1.58 times higher odds of child ASD than those with sufficient 25OHD. This association in the Nordic subsample was most prominent for ASD with ID, where 25OHD insufficiency was associated with 2.35 times higher odds of the outcome compared with 25OHD sufficiency. In a model with linear parameterization of 25OHD, each 25 nmol/L increase in maternal 25OHD was associated with 0.52 times lower odds of ASD with ID (Table 3). Generalized additive models were consistent with the linear parameterization, indicating a monotonic protective trend (Fig. S4). In sensitivity analyses of the 78% of participants with maternal 25OHD values measured in the 1st trimester, conclusions based on measured 25OHD values were broadly consistent with conclusions based on the standardized 25OHD values (Table S3).

**Table 3:** Associations of 25OHD concentrations and ASD in the Stockholm Youth Cohort

	Maternal 25OHD^a^		Neonatal 25OHD	
	Total sample: Number of exposed cases/odds ratio (95% CI)	Nordic^b^: Number of exposed cases/odds ratio (95% CI)	Total sample: Number of exposed cases/odds ratio (95% CI)	Nordic: Number of exposed cases/odds ratio (95% CI)
ASD				
<25 nmol/L 25OHD	46/1.67 (0.76, 3.67)	–	676/**1.33 (1.01, 1.74)**	421/**1.40 (1.04, 1.87)**
25–<50 nmol/L	114/1.19 (0.79, 1.78)	67/**1.58 (1.00, 2.49)**	573/1.11 (0.86, 1.43)	516/1.13 (0.86, 1.47)
≥50 nmol/L	289/Reference	261/Reference	150/Reference	142/Reference
25OHD increase of 25 nmol/L^c^	0.84 (0.60, 1.16)	**0.65 (0.43, 0.96)**	0.90 (0.78, 1.02)	**0.86 (0.74, 0.99)**
ASD without ID				
<25 nmol/L 25OHD	14/1.83 (0.64, 5.31)	–	428/**1.44 (1.06, 1.96)**	318/**1.48 (1.07, 2.05)**
25–<50 nmol/L	67/1.25 (0.79, 1.97)	48/1.47 (0.89, 2.44)	412/1.14 (0.86, 1.52)	383/1.16 (0.87, 2.05)
≥50 nmol/L	213/Reference	197/Reference	107/Reference	103/Reference
25OHD increase of 25 nnmol/L	0.80 (0.54, 1.17)	0.67 (0.43, 1.03)	0.90 (0.77, 1.04)	**0.85 (0.73, 1.00)**
ASD with ID				
<25 nmol/L 25OHD	32/1.62 (0.59, 4.44)	–	248/1.08 (0.72, 1.64)	103/1.15 (0.73, 1.82)
25–< 50 nmol/L	47/1.25 (0.67, 2.29)	19/**2.35 (1.14, 4.79)**	161/1.00 (0.69, 1.49)	133/1.00 (0.67, 1.53)
≥50 nmol/L	76/Reference	64/Reference	43/Reference	39/Reference
25OHD increase of 25 nmol/L	0.90 (0.54, 1.48)	**0.52 (0.27, 0.98)**	0.90 (0.73, 1.10)	0.87 (0.68, 1.10)

Estimates are derived from logistic regression models adjusted for year of birth, sample month, and maternal characteristics: psychiatric disorders, age, body mass index, smoking, nutritional supplement use, and region of origin

^a^Maternal 25OHD values are estimated from a prediction model that corrects for different gestational ages at sampling

^b^Estimates for the subset of children born to Nordic mothers are adjusted for all of above except for maternal region of origin

^c^Odds ratio associated with a linear increase of 25 nmol/L in maternal or neonatal 25OHD

### Neonatal vitamin D and ASD

For neonatal 25OHD, persons without ASD had median (IQR) concentrations of 26.6 nmol/L (17.3, 38.6), as compared with persons with ASD with ID [22.7 nmol/L (14.3, 34.1)] and persons with ASD without ID [26.9 nmol/L (18.8, 39.2)] (Table 2 Fig. 1c).

In adjusted models, compared with neonates with 25OHD ≥50 nmol/L, those with 25OHD <25 nmol/L had 1.33 times higher odds of ASD (Table 3). These associations were present for ASD without ID (1.43 times higher odds for <25 vs. ≥50 nmol/L) but not for ASD with ID. These results were also observed in the Nordic subsample. In a linear parameterization of 25OHD, an increase of 25 nmol/L was associated with 0.86 times the odds of ASD. Analyses of the matched-sibling sample (Fig. S4c) were similar but with reduced precision (OR and 95% CI per 1 SD linear increase in 25OHD: ASD: 0.86 [0.73, 1.01]; ASD without ID: 0.86 [0.69, 1.06]; ASD with ID: 0.88 [0.66, 1.16]), indicating that the results were unlikely to be due to familial confounding.

### Maternal and neonatal vitamin D and ASD

In adjusted analyses, having maternal and neonatal 25OHD values below the median was associated with elevated odds of ASD and ASD without ID as compared with having maternal and neonatal 25OHD both above the median (Table 4). Individuals with both maternal and neonatal 25OHD concentrations below the median had 1.75 times (95% CI: 1.08, 2.86) higher odds of ASD than persons with both maternal and neonatal 25OHD above the medians. Adjustment for maternal 25OHD did not influence the associations of neonatal 25OHD and ASD (Table S4), suggesting that the maternal and neonatal associations were largely independent of each other.

**Table 4:** Associations of maternal and neonatal 25OHD and ASD by presence or absence of intellectual disability in the Stockholm Youth Cohort. N of exposed ASD cases in each strata of analysis / odds ratio (95% confidence interval)

Outcome	Total sample *n* = 766	Nordic mothers^a^ *n* = 573
ASD		
Maternal below median, neonatal below median	131/**1.75 (1.08, 2.86)**	57/**1.94 (1.11, 3.41)**
Maternal below median, neonatal above median	60/1.48 (0.92, 2.40)	51/**1.78 (1.04, 3.06)**
Maternal above median, neonatal below median	51/1.17 (0.73, 1.89)	46/1.04 (0.62, 1.75)
Maternal above median, neonatal above median	98/Ref	93/Ref
ASD without ID		
Maternal below median, neonatal below median	73/**2.13 (1.24, 3.70)**	46/**1.99 (1.09, 3.63)**
Maternal below median, neonatal above median	40/1.50 (0.86, 2.59)	38/1.64 (0.91, 2.96)
Maternal above median, neonatal below median	39/1.11 (0.65, 1.89)	36/1.02 (0.57, 1.78)
Maternal above median, neonatal above median	74/Ref	72/Ref
ASD with ID		
Maternal below median, neonatal below median	58/1.24 (0.56, 2.72)	11/2.03 (0.76, 5.40)
Maternal below median, neonatal above median	20/1.46 (0.68, 3.08)	13/2.40 (0.95, 6.05)
Maternal above median, neonatal below median	12/1.20 (0.52, 2.65)	10/0.83 (0.32, 2.11)
Maternal above median, neonatal above median	24/Ref	21/Ref

Median sera 25OHD: 55.1 nmol/L; median neonatal 25OHD: 27.2 nmol/L

Estimates are derived from logistic regression models adjusted for year of birth, sample month, and maternal characteristics: psychiatric disorders, age, body mass index, smoking, nutritional supplement use, and region of origin

^a^Adjusted for all of above except for maternal region of origin

## Discussion

The present study in a Swedish population-based cohort examined vitamin D in prospectively collected maternal and neonatal blood samples and risk of ASD. Our results suggest that higher concentrations of 25OHD were associated with a modestly lower risk of ASD. In the overall sample, maternal 25OHD was not associated with ASD. However, in participants with Nordic mothers, higher maternal 25OHD was associated with lower odds of ASD. In analyses subtyped by co-occurring ID, higher maternal 25OHD was more associated with lower odds of ASD with ID, whereas higher neonatal 25OHD was more associated with lower odds of ASD without ID.

### Strengths and weaknesses

The present study features the largest number of ASD cases in a study of early life vitamin D and ASD. Vitamin D levels were measured from both maternal and neonatal blood, thus increasing the likelihood that the study reflects biological processes occurring during a temporal window of etiological relevance. The ASD case finding approach has been previously validated and occurred in the context of a universal healthcare system, increasing the likelihood that cases were identified. All study data were prospectively assessed, minimizing the potential for recall bias to influence results.

Nutritional epidemiology studies are often susceptible to confounding [41], since it is likely that nutritional patterns co-vary with other characteristics (e.g., healthful behaviors) that may influence risk of the outcome. Our study found that multiple characteristics differed between persons of different vitamin D status. Although our statistical models adjusted for a wide range of relevant covariates, results may still be influenced by confounding. The sibling analysis attempts to address this, through comparison of siblings who share early life environment and genetic background to a greater extent than unrelated individuals. Although our sibling analysis results did not reach conventional statistical significance, the point estimates were quite similar to our complete sample results, suggesting that familial environmental or genetic factors were unlikely to be responsible for observed associations. Still, confounding may be possible from a number of sources which could not be accounted for in the present study. For example, we did not measure concentrations of other nutrients in the neonatal blood samples, and it is possible that another nutrient that is highly correlated with vitamin D may in fact be responsible for the observed reduction in risk that is attributed to higher vitamin D concentrations. We previously reported that multivitamin supplement use at first antenatal visit was associated with reduced risk of ASD with ID [4], and while persons with multivitamin use had higher 25OHD concentrations, other nutrients are also likely elevated in such supplement users.

Another limitation of the present study is that it features a sample based in Sweden, thereby limiting the generalizability of results to other populations. Since vitamin D is influenced by sunlight exposure, the latitude of the study setting affects the possible exposure range of the study sample. We did not have adequate power to study the outcome of ASD among non-Nordic born mothers, which precluded any further analysis according to maternal region of origin. Our group has previously noted an elevated risk of ASD with ID for individuals born to mothers from Sub-Saharan and Northern Africa, Latin America, the Caribbean, and Southern Asia [42]. While non-Nordic born mothers and their children had notably lower levels of 25OHD compared with Nordic-born mothers, there are also complex cultural, environmental, and diagnostic factors that may be responsible for potential ASD risk differences between Nordic and non-Nordic groups in Sweden. This is also related to the next limitation of our study: although generalized additive models suggested a monotonically linear association between higher 25OHD and lower ASD risk, there were relatively few persons at the extreme values of 25OHD. This hinders examination of the upper range of values and potential thresholds of risk. For example, it is possible that increasing levels of 25OHD beyond a certain point may not actually further reduce ASD risk, but our study is unable to address such questions.

Caution must also be used in interpreting the cut-offs used in this study to denote deficiency and insufficiency. These cut-offs were set by the Institute of Medicine in reference to serum measurements in adults, while our study includes neonates and extrapolates serum equivalents from the concentrations measured in DBS. It is possible that different standards of deficiency and insufficiency should be applied to neonates compared to adults, although this issue has not been well studied. We observed that neonatal 25OHD levels were considerably lower than maternal levels measured during pregnancy. This is in line with long-standing observations that maternal 25OHD levels tend to be higher than, though positively correlated with, neonatal levels [43], as well as with recent observations from large cohort studies [13]. Previous studies using paired samples from mothers and their newborn children indicate that neonatal levels are on average lower than maternal levels and the prevalence of insufficiency is thus higher among neonates when the same cut-offs are used [21].

### Interpretation

Existing studies on the topic of 25OHD and ASD are few in number. Our findings agree with some of the existing literature but disagree with others. A recent analysis of birth trends of ASD cases in five countries found evidence for seasonality in Sweden and Finland. Specifically, the analysis found that children born in the fall months (i.e., conceived in the winter) had the highest risk of ASD and those born in the spring months (i.e., conceived in the summer) had the lowest risk of ASD [44]. Our findings that higher concentrations of perinatal/neonatal 25OHD are associated with lower risk of ASD is consistent with prior studies in the Netherlands [18, 21] and China [45], but disagree with overall null associations found in two studies from California [22, 23]. Laboratory differences are not a factor since the same laboratory was used for our study and the two California studies (the Early Markers for Autism (EMA) study and the CHARGE study). The California studies featured substantially higher vitamin D levels than in our study, as would be expected according to the geographical and sunlight differences between California and Sweden, and as such may explain differences in observed results. The EMA sample had a median neonatal 25OHD concentration of 84 nmol/L (controls); the CHARGE sample had a mean neonatal 25OHD concentration of 79.6 nmol/L; while our study had a median of 26.1 nmol/L. If there is a threshold effect of vitamin D on ASD risk, overall high levels in a sample could potentially mask such an effect. However, the California studies found racial/ethnic differences, with protective associations of higher 25OHD suggested for either ASD or developmental delay, but only in non-Hispanic whites. In our study, we assessed associations of 25OHD and ASD in analyses stratified by maternal origin, given the well-known differences by race/ethnicity in 25OHD. Consistent with the aforementioned findings above, evidence of a 25OHD-ASD association was observed in the subsample with mothers born in Nordic countries, a region that is generally associated with having lighter skin pigmentation. Even though our study was relatively large, the sample primarily consisted of mothers from Nordic countries. We had neither skin pigmentation data nor the statistical power to be able to stratify more finely by maternal origin to examine these differences further.

The observation that higher maternal 25OHD was more protective for ASD with ID (the more severe phenotype) but that higher neonatal 25OHD was more protective for ASD without ID, raises interesting questions about timing and critical windows for neurodevelopment. In the paired maternal-neonatal sample, we observed the lowest ASD risk for persons with both maternal and neonatal 25OHD above the median, and the highest risk for the group with both maternal and neonatal 25OHD below the median. Persons with maternal 25OHD below the median but neonatal 25OHD above the median had still elevated, but somewhat reduced risk, as compared with persons with both low maternal and neonatal 25OHD. Since fetal 25OHD is largely dependent on maternal levels [46], these results suggest that the ASD risk due to low 25OHD in early gestation may be slightly mitigated by increased 25OHD in later gestation. Therefore, 25OHD may have more substantial influence on ASD risk earlier in gestation as opposed to later, with lower levels earlier on increasing risk for a more severe phenotype of ASD with ID, and lower levels later on increasing risk for the less severe phenotype of ASD without ID. Only a few studies have examined critical windows for vitamin D for neurodevelopment. In experimental studies using rats, developmental vitamin D deficiency is known to result in enlarged lateral ventricles in neonates [47]. However, adding vitamin D back into the diet at birth partially ameliorated these structural changes [48]. O’Loan et al. compared behavioral phenotypes of rats exposed to different periods of vitamin D deficiency during gestation [49]. Rats exposed to late deficiency (a vitamin D-deficient maternal diet during pregnancy), and full deficiency (a vitamin D-deficient maternal diet both pre-conception and during pregnancy) exhibited increased hyperlocomotion in response to treatment with the NDMA-receptor antagonist MK-801. Interestingly, rats with early deficiency (a vitamin D-deficient maternal diet pre-conception, but with a replete diet during pregnancy) did not exhibit such abnormal behavior, suggesting that prompt prenatal intervention may reduce adverse outcomes.

It should be noted that although we found that 25OHD was associated with ASD in general regardless of co-occurring ID, it may be possible that 25OHD influences neurodevelopmental processes in a way that is not specific to ASD. A recent meta-analysis suggests that increased prenatal 25OHD is associated with better cognitive development and lower risk of ADHD [50]. Lower neonatal vitamin D status has also been associated with increased odds of schizophrenia [29]. There is robust evidence from animal studies that vitamin D is involved in a range of processes relevant to the developing brain, including cell differentiation, cytokine regulation, neurotransmitter synthesis, calcium signaling, anti-oxidant activity, and gene/protein expression governing neuronal physiology [51]. Thus, it is plausible that vitamin D may influence risk of ASD and other neurodevelopmental conditions through any of these mechanisms. Increasing evidence suggests that the placenta is instrumental in mediating the manifold effects of vitamin D [46]. The placenta expresses the components necessary for vitamin D signaling including the vitamin D receptor (VDR), retinoid X receptor (RXR), and the cytochrome P450 enzymes CYP27B1 and CYP24A1. Vitamin D has immunomodulatory properties, generally reducing adaptive immune activity and promoting innate immune activity [52]. Epidemiological studies have shown that maternal hospitalization with infection during pregnancy is associated with increased risk of ASD in offspring [53, 54]. This is consistent with animal models of maternal immune activation that show ASD-related phenotypes resulting from maternal injection with lipopolysaccharide (as a bacterial infection analogue) or poly(I:C) (as a viral infection analogue) [55]. Interestingly, administration of calcitriol to poly(I:C)-exposed pregnant mice prevents the ASD-related phenotypes in offspring [56]. In rats, challenge of vitamin D-deficient placentas with poly(I:C) resulted in higher production of IL-6 and IL-1B in male fetuses [57]. In humans, elevated levels of maternal IL-6 [58] and neonatal IL-1B [59] have been associated with increased ASD risk. Further studies are required to understand if the influence of early life vitamin D exposure on maternal-fetal immune responses constitutes a plausible mechanism linking early life vitamin D exposure to risk for ASD in humans.

## Conclusions

Even with mounting epidemiological evidence from our study and others linking early life vitamin D to ASD, it is premature to conclude that a causal link exists, since all studies to date have been observational in nature. Ethically designed randomized controlled trials of vitamin D supplementation during pregnancy would allow for a clear answer.

## Supplementary information

Supplement
