# Supplementary material for: Developmental vitamin D and autism spectrum disorders: findings from the Stockholm Youth Cohort
Source: Mol Psychiatry. 2019 Nov 6;26(5):1578–88. doi: 10.1038/s41380-019-0578-y (PMC7200274; doi:10.1038/s41380-019-0578-y)
Supplement: Supplementary file 1 — Supplement [file 41380_2019_578_MOESM1_ESM.docx]

**Supplement**

Neonatal dried blood spot sampling

Sampling for the neonatal analysis and sibling-controlled neonatal analysis is shown in **Figure S1**. In 2014, we selected 3,915 participants from the source population (SYC birth cohorts 1996-2000) for neonatal blood collection, comprising: (A) a random sample of 1,999 SYC participants (thus including some ASD cases); (B) all 1,496 participants in the source population with an ASD diagnosis as of December 31, 2007 (together A and B represent a case-cohort sample); and (C) 456 unaffected siblings of ASD cases -- in other words, siblings without an ASD diagnosis as of December 31, 2007. After exclusion of participants due to non-collection of samples and laboratory failures, 3,405 participants (87.0% of the original sample) with valid vitamin D measurements were retained. In 2018, we obtained updated ASD diagnosis data current as of December 31, 2016. With the update, there were 98 new ASD cases: 54 members of the random sample previously without an ASD diagnosis, and 44 siblings who were previously unaffected. The unaffected persons from the random sample and all cases with measured vitamin D were analyzed as a case-control study of 1,399 ASD cases and 1,607 controls. The ASD cases with unaffected matched siblings (357 cases and 364 unmatched siblings) were analyzed as a sibling-matched case-control study, to account for potential familial confounding.

Sera sampling

Sampling for the maternal analysis is shown in **Figure S2**. From the random sample and ASD cases described in (A) and (B) above, 1,023 persons born 1997-2000 were randomly selected for maternal sera collection. These 1,023 persons included 425 ASD cases and 598 unaffected persons. After update of ASD diagnoses in 2016, 24 formerly unaffected persons converted to ASD status, resulting in a maternal analytic sample of 449 ASD cases and 574 unaffected persons. Of these, 340 ASD cases and 426 unaffected persons also had a neonatal vitamin D measurement.

**Figure S1: Derivation of neonatal sample and neonatal sibling sample.**


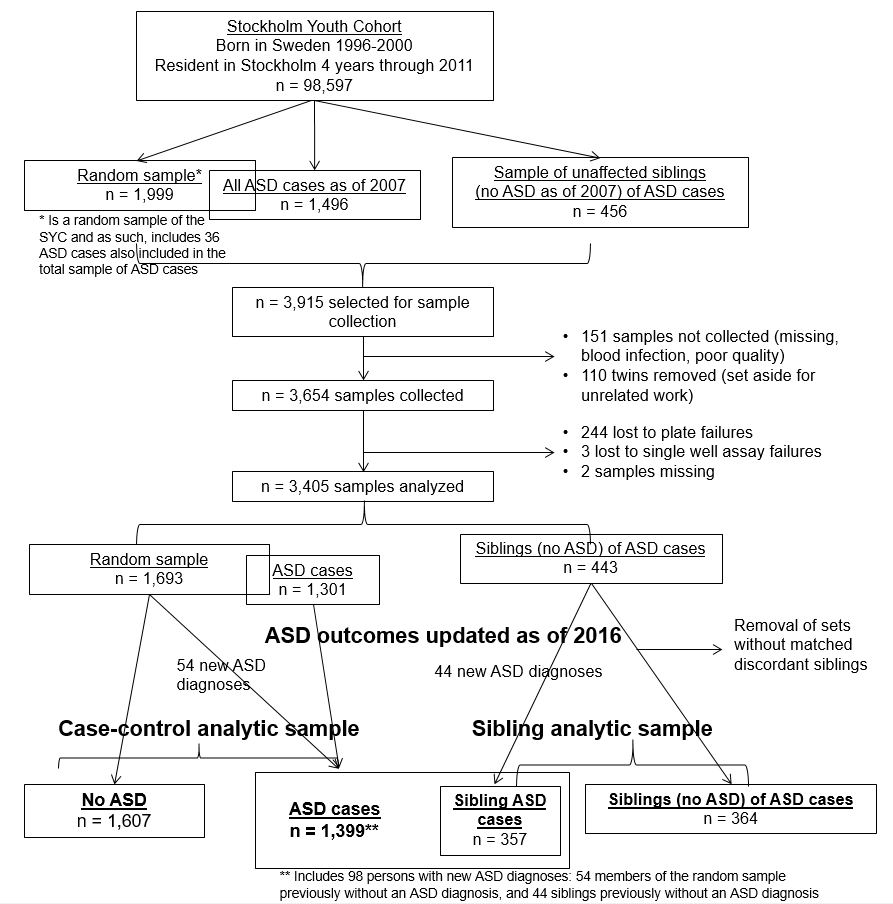


**Figure S2: Derivation of maternal sample**


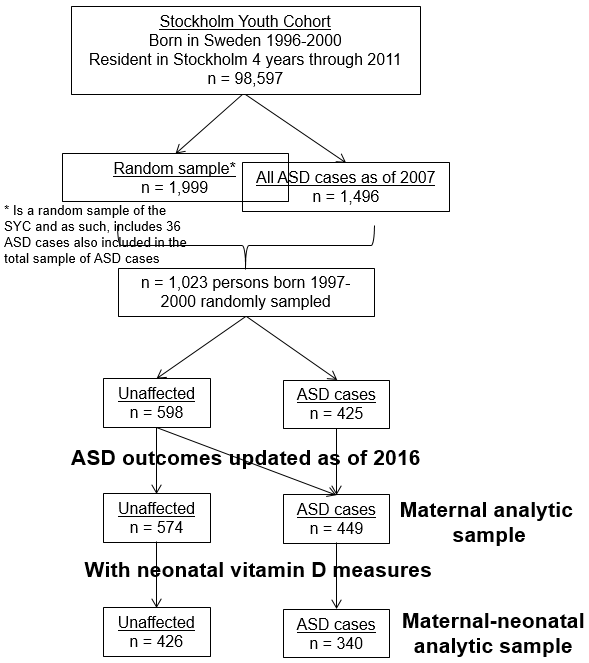


Standardization of maternal 25OHD values.

There was variation in time of maternal sera sampling (median 10.9 weeks gestation, with interquartile range: 9.3, 13.0 weeks) (**Figure S3**). Comparing women with very different times of sampling (e.g., 8 weeks versus 35 weeks) is not likely to yield valid conclusions for two reasons. First, 25OHD concentrations vary with season, so a high value at a given point in time does not indicate that the concentration will be high at all other points in time. Second, any conclusions about the association of maternal 25OHD with child ASD without accounting for such variation in sampling time may be misguided given that animal models suggest there are developmental windows for which vitamin D deficiency have greater impact. Therefore, to ensure comparability, we used cosinor modeling to standardize 25OHD values at gestational age of 10.9 weeks for each observation. A simple depiction of the standardization process is shown in **Figure S3a** where the goal is to, based on the available data, estimate what a person’s 25OHD value would have been had she been sampled at 10.9 weeks. 10.9 weeks was chosen as the standardization time since it was the observed median and mode time of sampling. Since the majority of the data was centered at 10.9 weeks (80% of the participants had sampling times within 4 weeks of this timepoint), this reduces the possibility of extrapolating too far out for the data.

Cosinor modeling is useful for modeling stationary sinusoidal patterns of a seasonal nature (i.e., that would not greatly vary from year to year). Cosinor models have been previously applied to model seasonal variation in 25OHD in Norway.^1^ A standard cosinor model may have the following form:

$$y\left( t \right)= \beta_{0} +\beta_{1}\times\cos\frac{2\pi t}{T}+\beta_{2}\sin\frac{2\pi t}{T}$$

where:
*y(t)* is the outcome at time *t* and *t*  = the underlying time-scale variable

*T* = length of time of one period. *T* was set to 365 days in order to fit a yearly seasonal model.

The full modeling approach was as follows: Step 1) randomly split the analytic sample into a training set (80%) and test set (20%); Step 2) in the training set, use a random forests model with cosinor terms as described above and covariates, with 5-fold cross validation, to estimate maternal 25OHD. The covariates were: month of sera sampling; gestational age at time of sampling; plate number; and maternal characteristics: age, body mass index, anemia, smoking, nutritional supplement use, and region of origin. Step 3) use the model developed in Step 2 to predict maternal 25OHD in the test set and validate against actual observed 25OHD values in the test set. Development of the model was performed in R using the *caret* package.^2^

The 25OHD values standardized to 10.9 weeks collection were correlated at r = 0.96 with the measured 25OHD (**Figure S3b**). Overall, standardized values were very similar with measured values (**Figure S3c**). The median (interquartile range) difference between measured and standardized 25OHD values was -1.1 nmol/L (-6.2, 4.8). In total, 72.5% of observations had a standardized 25OHD value within 10 nmol/L of the measured value. This is in line with past studies that yielded 57%,^3^ 59%,^1^ and 74%^4^ of predicted 25OHD values within 10 nmol/L of observed values. Moreover, plots of standardized 25OHD (**Figure S4d,e**) vs. measured 25OHD (**Figure S3c,e**) by maternal origin and child ASD status were similar in nature, indicating that the standardized values were in range of expectations based on the measured values. After validating that the prediction model performed well in the test set, we used the model to estimate in the entire dataset the 25OHD values for all participants at 10.9 weeks gestational age. This standardized 25OHD was therefore the main predictor variable of interest for the maternal sera analysis.

**Figure S3**: Standardized sera 25OHD. A) Schematic of standardization of sera sampling time. Left histogram shows observed gestational age at time of sera sampling; right histogram shows gestational age standardized to 10.9 weeks; B) Correlation of measured vs. predicted sera 25OHD values in the test set; C) Measured vs. predicted sera 25OHD values by date of sampling in the test set; D) and E): standardized sera 25OHD by maternal country of origin and child ASD status

| A 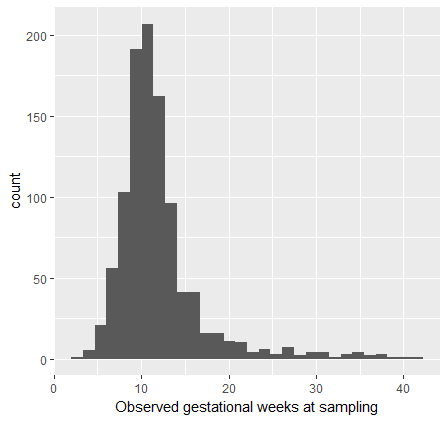 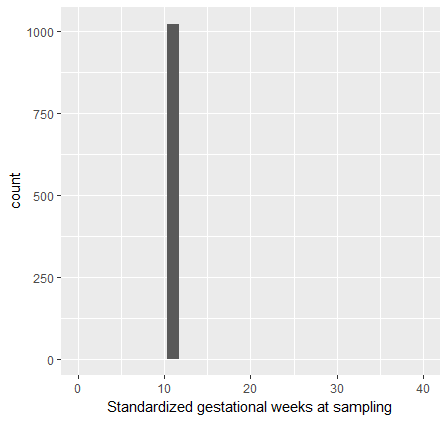 | |
| --- | --- |
| B  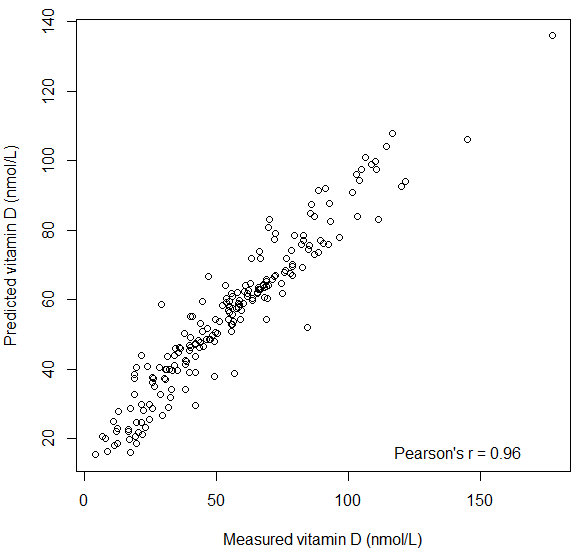 | C  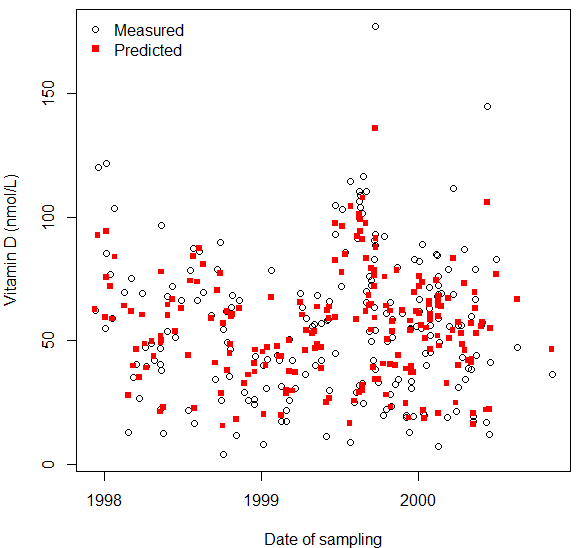 |
| D 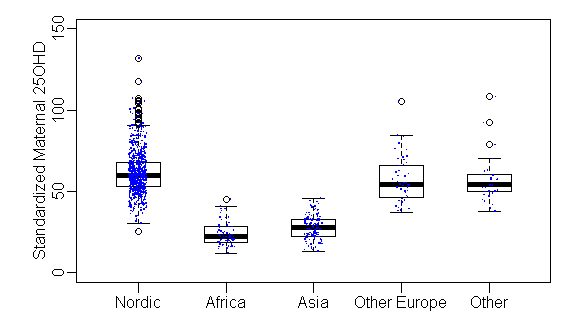 | E 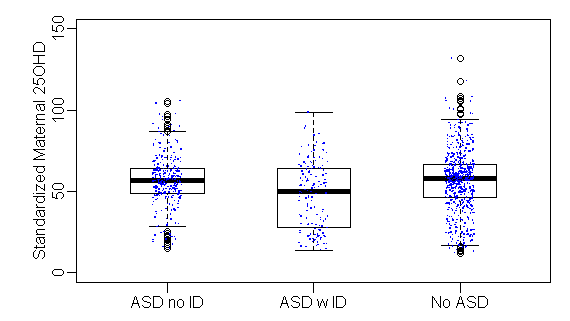 |

**Table S1**: Maternal analytic sample and maternal-neonatal analytic sample characteristics

|  | Maternal sample | | Maternal-neonatal sample | |
| --- | --- | --- | --- | --- |
|  | No ASD n = 574 | ASD n = 449 | No ASD n = 426 | ASD n = 340 |
| Outcome  ASD no ID  ASD with ID  No ASD | -  -  574 (100) | 294 (65.5)  155 (34.5) - | -  -  426 (100) | 226 (66.5)  114 (33.5)  - |
| Season of birth  Spring (Mar-May)  Summer (Jun-Aug)  Fall (Sep-Nov)  Winter (Dec-Feb) | 139 (24.2) 154 (26.8) 140 (24.4) 141 (24.6) | 94 (20.9) 123 (27.4) 133 (29.6) 99 (22.0) | 48 (11.3) 142 (33.3)  134 (31.5) 102 (23.9) | 31 (9.1) 115 (33.8) 125 (36.8) 69 (20.3) |
| Male | 305 (53.1) | 346 (77.1) | 219 (51.4) | 269 (79.1) |
| Maternal age, mean (SD) | 30.7 (4.9) | 30.7 (5.2) | 30.7 (4.9) | 30.7 (5.3) |
| Maternal BMI  Normal  Underweight  Overweight  Obese  Missing | 269 (46.9)  9 (1.6) 83 (14.5) 26 (4.5)  187 (32.6) | 171 (38.1)  8 (1.8)  79 (17.6)  30 (6.7) 161 (35.9) | 203 (47.7)  9 (2.1) 65 (15.3) 20 (4.7) 129 (30.3) | 131 (38.5) 8 (2.4) 62 (18.2) 23 (6.8)  116 (34.1) |
| Maternal smoking | 49 (8.5) | 37 (8.2) | 38 (8.9) | 26 (7.6) |
| Maternal supplementation  Multivitamins  Iron only  Folic acid only  Iron and folic acid  None of the above | 123 (21.4)  148 (25.8)  4 (0.7)  68 (11.8)  231 (40.2) | 77 (17.1) 125 (27.8)  6 (1.3)  49 (10.9)  192 (42.8) | 88 (20.7)  106 (24.9)  3 (0.7) 54 (12.7) 175 (41.1) | 63 (18.5) 96 (28.2) 6 (1.8) 34 (10.0) 141 (41.5) |
| Maternal psychiatric history | 184 (32.1) | 210 (46.8) | 138 (32.4) | 164 (48.2) |
| Maternal birth country  Nordic  Africa  Asia  Other Europe  Other | 438 (76.3) 33 (5.7) 61 (10.6) 22 (3.8)  20 (3.5) | 328 (73.1)  34 (7.6)  51 (11.4)  23 (5.1) 13 (2.9) | 326 (76.5) 24 (5.6) 43 (10.1) 15 (3.5) 18 (4.2) | 247 (72.6)  31 (9.1) 36 (10.6)  15 (4.4) 11 (3.2) |

**Table S2**: Comparison of persons included in the neonatal analysis vs. those excluded due to non-collection and laboratory failures

|  |  | |
| --- | --- | --- |
|  | Included  n = 3,370 | Excluded  n = 545 |
| Outcome  ASD no ID  ASD with ID  No ASD | 947 (28.1) 452 (13.4) 1971 (58.5) | 124 (22.8) 87 (16.0)  334 (61.3) |
| Season of birth  Spring (Mar-May)  Summer (Jun-Aug)  Fall (Sep-Nov)  Winter (Dec-Feb) | 818 (24.3)  920 (27.3)  851 (25.3)  781 (23.2) | 230 (42.2)  90 (16.5)  74 (13.6)  151 (27.7) |
| Male | 2047 (60.7) | 327 (60.0) |
| Gestational age at birth in days, mean (SD) | 278.7 (13.2) | 274.4 (17.4) |
| Maternal age, mean (SD) | 30.1 (5.1) | 30.6 (5.1) |
| Maternal BMI  Normal  Underweight  Overweight  Obese  Missing | 1599 (47.4)  83 (2.5)  594 (17.6)  223 (6.6)  871 (25.8) | 219 (40.2)  2 (0.4)  71 (13.0)  29 (5.3)  224 (41.1) |
| Maternal smoking | 356 (10.6) | 44 (8.1) |
| Maternal supplementation  Multivitamins  Iron only  Folic acid only  Iron and folic acid  None of the above | 413 (12.3)  1066 (31.6)  35 (1.0)  289 (8.6)  1564 (46.4) | 76 (13.9)  140 (25.7)  6 (1.1)  55 (10.1)  268 (49.2) |
| Maternal neuropsychiatric conditions  ASD  ID  Psychiatric history | 56 (1.7)  16 (0.5)  1407 (41.8) | 13 (2.4)  3 (0.6)  205 (37.6) |
| Maternal birth country  Nordic  Africa  Asia  Other Europe  Other | 2593 (76.9)  227 (6.7)  313 (9.3)  131 (3.9)  106 (3.1) | 381 (69.9)  37 (6.8)  79 (14.5)  32 (5.9)  16 (2.9) |
| Parental education (highest) | 1268 (37.6) | 223 (40.9) |
| Family income quintile (highest) | 648 (19.2) | 84 (15.4) |

**Figure S4: Generalized additive model fits of the association of 25OHD and ASD**

| **A 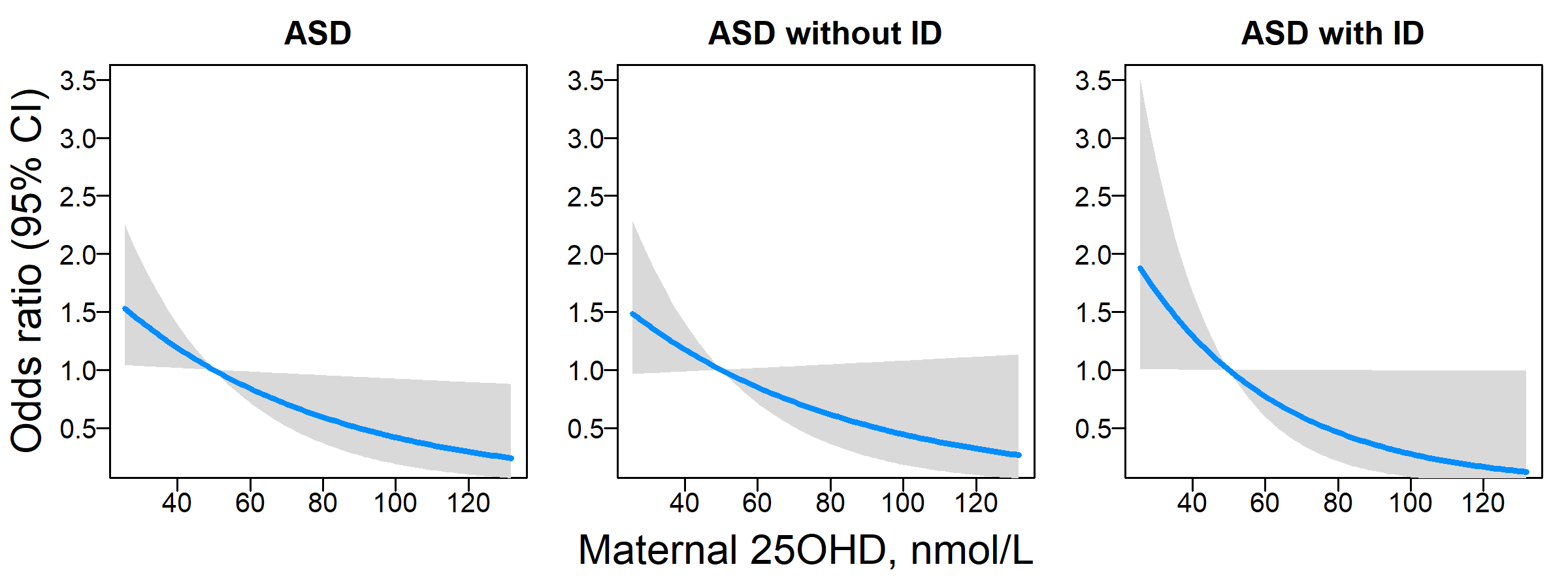** |
| --- |
| **B 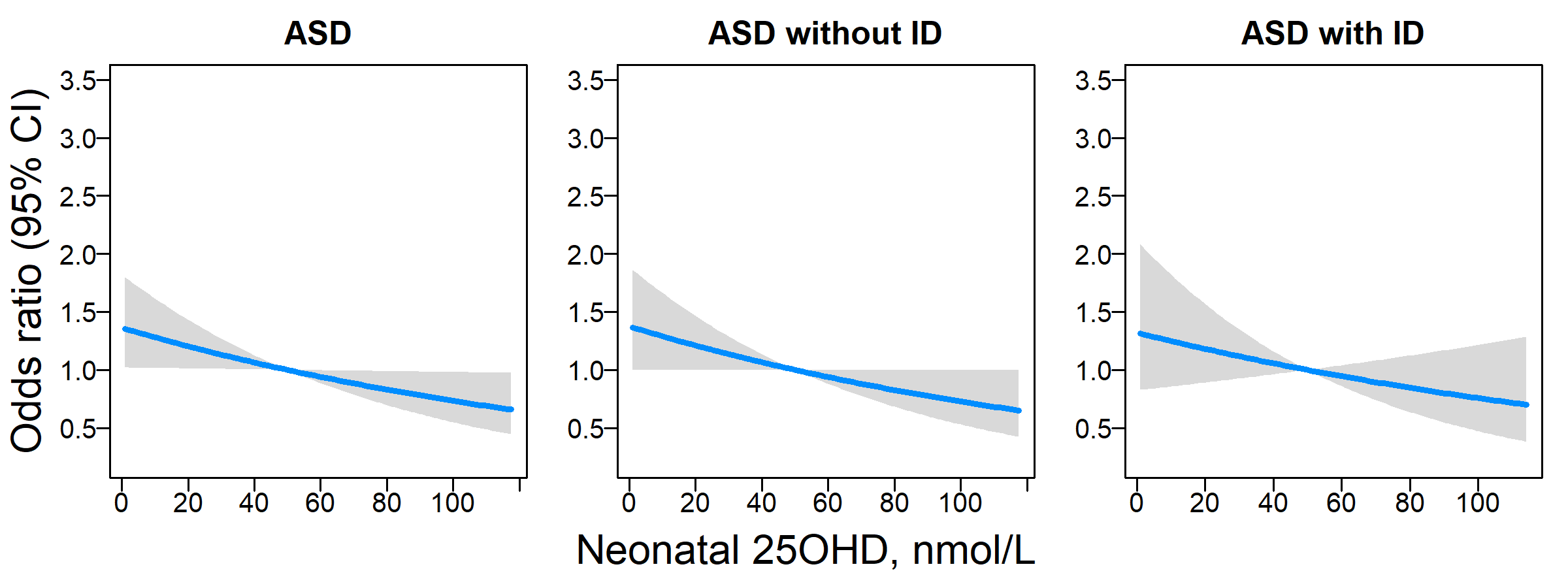** |
| **C 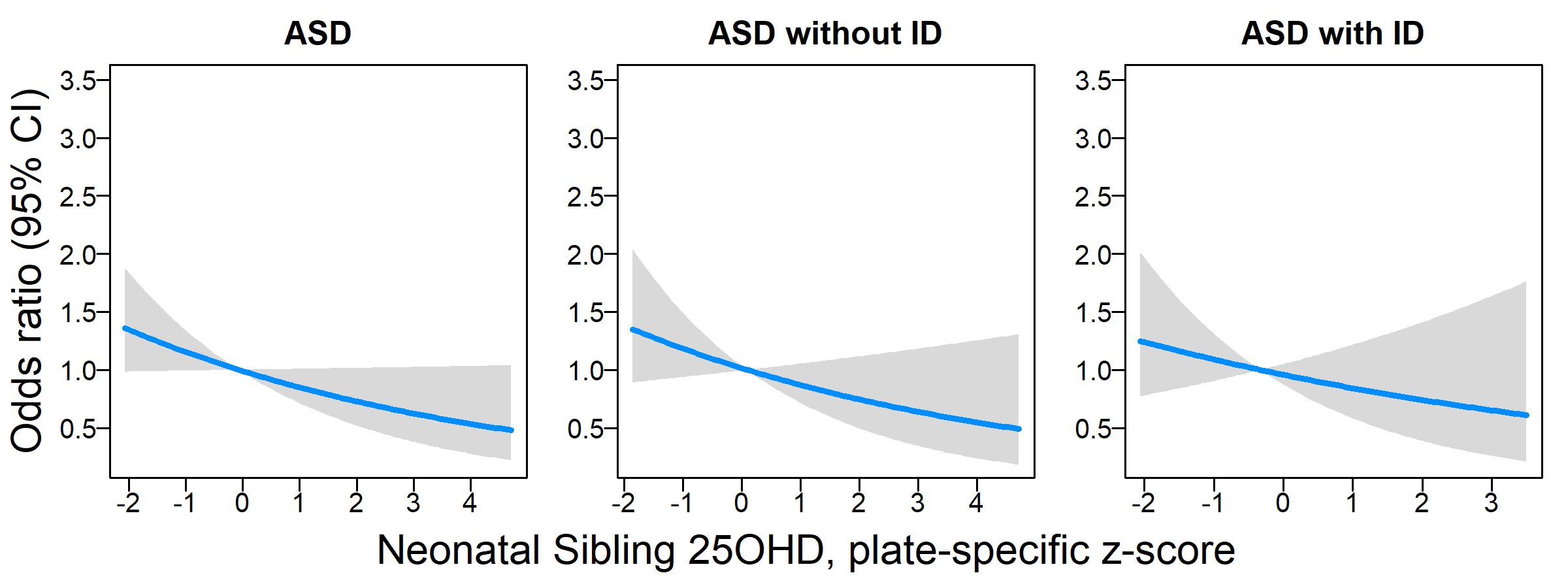** |

**Table S3**: Sensitivity analysis: associations of first-trimester measured sera 25OHD concentrations and ASD by presence or absence of intellectual disability in the Stockholm Youth Cohort. N of exposed ASD cases in each strata of analysis / odds ratio (95% confidence interval).

| Outcome | Total sample measured in 1^st^ trimester (N=787) | Nordic mothers measured in 1^st^ trimester** (N=626) |
| --- | --- | --- |
| ASD  <25 nmol/L  25-<50  50+  25OHD increase of 25 nmol/L**** | 49 / 1.62 (0.87, 3.03)  113 / 1.24 (0.87, 1.76)  177 / REF  **0.82 (0.69, 0.97)** | 13 / 1.77 (0.70, 4.59)  92 / 1.43 (0.97, 2.10)  161 / REF  **0.76 (0.62, 0.93)** |
| ASD without ID  <25 nmol/L  25-<50  50+  25OHD increase of 25 nnmol/L | 18 / 1.01 (0.47, 2.15)  84 / 1.27 (0.87, 1.87)  134 / REF  0.85 (0.70, 1.03) | 8 / 1.57 (0.53, 4.56)  72 / 1.44 (0.95, 2.19)  123 / REF  **0.77 (0.61, 0.95)** |
| ASD with ID  <25 nmol/L  25-<50  50+  25OHD increase of 25 nmol/L | **31 / 3.30 (1.40, 7.78)**  29 / 1.23 (0.68, 2.20)  43 / REF  **0.73 (0.54, 0.97)** | 5 / 2.87 (0.75, 9.93)  20 / 1.47 (0.75, 2.84)  38 / REF  **0.67 (0.47, 0.94)** |

* Logistic regression model is adjusted for year of birth, sera sample month, maternal psychiatric disorders, maternal age, maternal body mass index, maternal smoking, nutritional supplement use, and maternal region of origin.

** Adjusted for all of above except for maternal region of origin

**** Odds ratio associated with a linear increase of 25 nmol/L in maternal 25OHD

**Table S4**: Associations of neonatal 25OHD concentrations and ASD in the maternal-neonatal sample, without and with adjustment for maternal 25OHD

| Outcome | Total sample, not adjusted for maternal 25OHD* (n=766) | Total sample, adjusted for maternal 25OHD* (n=766) | Nordic subsample, not adjusted for maternal 25OHD** (n=573) | Nordic subsample, adjusted for maternal 25OHD** (n=573) |
| --- | --- | --- | --- | --- |
| ASD  Neonatal 25OHD increase of 25 nmol/L  Maternal 25OHD increase of 25 nmol/L | 0.89 (0.67, 1.17)  - | 0.92 (0.69, 1.22)   0.76 (0.48, 1.19) | 0.81 (0.59, 1.11)  - | 0.86 (0.62, 1.18)   0.66 (0.39, 1.12) |
| ASD without ID  Neonatal 25OHD increase of 25 nnmol/L  Maternal 25OHD increase of 25 nmol/L | 0.81 (0.59, 1.11) | 0.86 (0.61, 1.18)   0.67 (0.39, 1.11) | 0.78 (0.55, 1.10)  - | 0.82 (0.57, 1.17)   0.68 (0.37, 1.21) |
| ASD with ID  Neonatal 25OHD increase of 25 nmol/L  Maternal 25OHD increase of 25 nmol/L | 1.18 (0.74, 1.84)  - | 1.19 (0.75, 1.87)   0.91 (0.45, 1.79) | 0.97 (0.54, 1.68)  - | 1.07 (0.59, 1.89)   0.43 (0.16, 1.10) |

* Logistic regression model is adjusted for year of birth, sera sample month, maternal psychiatric disorders, maternal age, maternal body mass index, maternal smoking, nutritional supplement use, and maternal region of origin.

** Adjusted for all of above except for maternal region of origin

Works cited

1. Degerud E, Hoff R, Nygard O, et al. Cosinor modelling of seasonal variation in 25-hydroxyvitamin D concentrations in cardiovascular patients in Norway. *Eur J Clin Nutr.* 2016;70(4):517-522.

2. Kuhn M. Building predictive models in R using the caret package. *Journal of statistical software.* 2008;28(5):1-26.

3. Sachs MC, Shoben A, Levin GP, et al. Estimating mean annual 25-hydroxyvitamin D concentrations from single measurements: the Multi-Ethnic Study of Atherosclerosis–. *The American journal of clinical nutrition.* 2013;97(6):1243-1251.

4. Bolland MJ, Chiu WW, Davidson JS, et al. The effects of seasonal variation of 25-hydroxyvitamin D on diagnosis of vitamin D insufficiency. *The New Zealand Medical Journal (Online).* 2008;121(1286).
